# Haemoglobin variants and *Plasmodium falciparum* malaria in children under five years of age living in a high and seasonal malaria transmission area of Burkina Faso

**DOI:** 10.1186/1475-2875-11-154

**Published:** 2012-05-04

**Authors:** Edith C Bougouma, Alfred B Tiono, Alphonse Ouédraogo, Issiaka Soulama, Amidou Diarra, Jean-Baptiste Yaro, Espérance Ouédraogo, Souleymane Sanon, Amadou T Konaté, Issa Nébié, Nora L Watson, Megan Sanza, Tina JT Dube, Sodiomon B Sirima

**Affiliations:** 1 Centre National de Recherche et de Formation sur le Paludisme, Ouagadougou, Burkina Faso; 2 Groupe de Recherche et Action en Santé, Ouagadougou, Burkina Faso; 3 The EMMES Corporation Rockville, Maryland, USA

**Keywords:** *Plasmodium falciparum*, Malaria, Haemoglobin abnormalities, Children, Epidemiology, Burkina Faso

## Abstract

**Background:**

Genetic factors play a key role in determining resistance/susceptibility to infectious disease. Susceptibility of the human host to malaria infection has been reported to be influenced by genetic factors, which could be confounders if not taken into account in the assessment of the efficacy of interventions against malaria. This study aimed to assess the relationship between haemoglobin genotypes and malaria in children under five years in a site being characterized for future malaria vaccine trials.

**Methods:**

The study population consisted of 452 children living in four rural villages. Hb genotype was determined at enrolment. Clinical malaria incidence was evaluated over a one-year period using combined active and passive surveillance. Prevalence of infection was evaluated via bi-annual cross-sectional surveys. At each follow-up visit, children received a brief clinical examination and thick and thin blood films were prepared for malaria diagnosis. A clinical malaria was defined as *Plasmodium falciparum* parasitaemia >2,500 parasites/μl and axillary temperature ≥37.5°C or reported fever over the previous 24 hours.

**Results:**

Frequencies of Hb genotypes were 73.2% AA; 15.0% AC; 8.2% AS; 2.2% CC; 1.1% CS and 0.2% SS. Prevalence of infection at enrolment ranged from 61.9%-54.1% among AA, AC and AS children. After one year follow-up, clinical malaria incidence (95% CI) (episodes per person-year) was 1.9 (1.7-2.0) in AA, 1.6 (1.4-2.1) in AC, and 1.7 (1.4-2.0) in AS children. AC genotype was associated with lower incidence of clinical malaria relative to AA genotype among children aged 1–2 years [rate ratio (95% CI) 0.66 (0.42-1.05)] and 2–3 years [rate ratio (95% CI) 0.37 (0.18-0.75)]; an association of opposite direction was however apparent among children aged 3–4 years. AS genotype was associated with lower incidence of clinical malaria relative to AA genotype among children aged 2–3 years [rate ratio (95% CI) 0.63 (0.40-1.01)].

**Conclusions:**

In this cohort of children, AC or AS genotype was associated with lower risk of clinical malaria relative to AA genotype only among children aged one to three years. It would be advisable for clinical studies of malaria in endemic regions to consider haemoglobin gene differences as a potentially important confounder, particularly among younger children.

## Background

Malaria remains one of the most important causes of morbidity and mortality in endemic areas, primarily affecting children under five years of age [1]. The highest death burden occurs in young children who have not yet developed protective immune mechanisms against the parasite. A minority of children appear to have a natural biological advantage thought to partially impede parasite growth [2].

Malaria is a complex disease that depends on many host genetic factors [3]. Indeed, resistance to *Plasmodium falciparum* is an important adaptive trait of human populations living in endemic areas [4]. Haemoglobin S (HbS) has become a stable polymorphism within malaria-endemic regions, associated with a limited life expectancy among homozygous individuals who suffer from sickle cell disease, and an extended life expectancy of heterozygous individuals who are more likely to evade malaria [5-7]. HbAS is widely known to confer significant protection from severe and uncomplicated malaria [6-12] although underlying mechanisms not precisely defined. Similar protection afforded by haemoglobin C (HbC) was more recently demonstrated although findings are less conclusive. Clinical studies performed in Nigeria and Mali has found no protection [13-15], while other Malian study and Burkina study indicated an association between HbAC and clinical malaria [16,17].

Several innate or immune mechanisms have been hypothesized to explain malaria-protective effects of HbS or HbC [2,18-20] Erythrocytes containing HbS or HbC may impede parasite growth and replication relative to normal red cells when subject to low oxygen tensions [18]. Protein targets of specific antibodies may be more rapidly exposed in HbS-containing red blood cells [21] resulting in an enhanced immune response to infection [18,22]. It is also possible that unknown innate protective processes may up-regulate the malaria-specific immune response [23] or enhance nonspecific immunity to malaria [24].

The comparison of malaria indicators among populations with different genetic backgrounds that are uniformly exposed to the same parasite strains is one approach to the study of human heterogeneities in response to the infection [9,17]. To characterize malaria risk in the population residing in the malaria vaccine trial site in Saponé, Burkina Faso, a haemoglobin genotyping study was conducted in children under five years of age living in this malaria endemic region. The study aimed to describe the relationship between abnormal haemoglobin genotypes and malaria in children under five years in a site being characterized for future malaria vaccine trials. Age-specific patterns of association were hypothesized to reflect the development of acquired immunity throughout early childhood.

## Methods

### Study area and period

**Figure 1:**
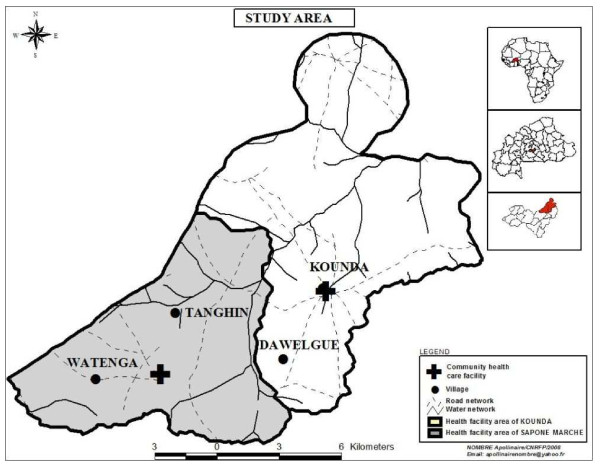
Study site in Burkina Faso.

The study was conducted in four villages (Dawelgue, Tanghin, Kounda, and Watenga) in the Saponé Health District, located 50 km south-west of Ouagadougou, the capital city of Burkina Faso, in the Bazega province (Figure 1). The region is populated almost exclusively by the Mossi ethnic group, and farming is the main subsistence activity. The area has a rainy season lasting from June to October, corresponding to the high malaria transmission season, and a long dry season from November to May. The main malaria vectors are *Anopheles gambiae, Anopheles arabiensis*, and *Anopheles funestus*. The entomological inoculation rate (EIR) in 2001 was estimated at 0.3 and 44.4 infective/bites/person/month during the dry and rainy seasons, respectively in study area [25]. A demographic surveillance system (DSS) for monitoring vital events has been operating in the villages since 2002. 

Enrolment was initiated on 23 January, 2007 and the final study visit for the last subject was completed on 29 February, 2008.

### Study participants

The study population consisted of children under five years of age identified from the above four villages whose parents were also resident in the study area. The sample was identified from a census list generated from the DSS census list of all children under five years living in the study region. Children were recruited approximately equally among age groups as follows: nought to 11 months, 12–23 months, 24–35 months, 35–47 months and 48–49 months.

### Study design and sample collection

Exclusion criteria from the cohort included major congenital defects, any chronic disease, or anaemia defined as measured haemoglobin <6 g/dl. Inclusion criteria were as follows: i) written/thumb-printed informed consent obtained from the parent or legal guardian of the child, ii) permanent resident in the study area and expected to remain so for at least one year following enrolment, iii) age between nought and five years.

Prevalence of infection and malariometric indices were evaluated during bi-annual cross-sectional surveys within the low and high transmission seasons. The incidence of clinical malaria was documented through active and passive surveillance from the first survey through one year follow-up.

### Cross-sectional surveys

Each cross-sectional survey consisted of a brief clinical examination and collection of blood by finger prick. The sampled blood was used for thick and thin blood film preparation for malaria microscopic examination. Haemoglobin measurements were done using a HemoCue machine. A venous blood sample of 5 ml was additionally collected at the first survey for haemoglobin genotyping.

### Active case detection

During the 12-month period of longitudinal surveillance, each child was visited twice per week by nurses living in the villages. The visit consisted of a brief history and clinical examination. Among children with an axillary temperature ≥ 37.5°C or reported history of fever in the past 24 hours, finger-prick blood samples were collected for haemoglobin measurement and malaria microscopic examination. Subjects with fever and positive rapid diagnostic test (RDT, OptiMAL) were recommended to visit the medical centre for treatment with artemether-lumefantrine.

### Passive case detection

Parents of the children were encouraged to report to the nearest community clinic or hospital any time their child showed a sign of sickness in between home visits. Each child in attendance at either health facility received a brief clinical examination; any child with an axillary temperature ≥ 37.5°C or reported history of fever in the past 24 hours was tested for malaria and treated accordingly.

### Parasitological diagnosis

Blood slides from either cross-sectional or longitudinal surveys were stained with Giemsa for microscopic identification of the *Plasmodium* species and determination of parasite density. Thick and thin blood films were air-dried; thin blood films were fixed with methanol, and both were stained with 3% Giemsa. One hundred high power fields were examined and the number of malaria parasites of each species and stage were recorded. The number of parasites per microlitre of blood was calculated by assuming that there were 20 white blood cells per high-power field and a fixed cell count of 8,000 per μl. Each film was read twice by two experienced technicians and a third reading was undertaken if discrepancies exceeding 30% occurred between the two readers.

### Haemoglobin typing

DNA was extracted using a QIAGEN kit (QIAamp DNA blood mini kit) and the haemoglobin was typed by polymerase chain reaction-restriction fragment length polymorphism (PCR-RFLP). Briefly, DNA samples were amplified using a 5’-AGG AGC AGG GAG GGC AGG A-3’ forward primer and a 5’-TCC AAG GGT AGA CCA CCA GC-3’ reverse primer. The 358 base pair bp fragment obtained was digested with MnlI for discrimination between HbAA (173 pb, 109 pb, and 60pb), HbSS/HbCC andHbSC (173 pb, 109 pb, and 76 pb), HbAS/HbAC (173 pb, 109 pb, 76 pb and 60). A second digestion with DdeI allowed for further discrimination for ambiguous results between HbSS (331 pb), HbCC (201 pb and 130 pb), HbSC (130 pb, 201 pb and 331 pb), HbAS (130 pb, 201 pb and 331 pb) and HbAC (201 pb and 130 pb). All digestion was carried out for three hours at 37°C and the products were run on a 3% agarose gel [17].

### Malariometric indices

Malariometric indices were evaluated during the low and high transmission seasons using measures obtained from the biannual surveys. A clinical *P. falciparum* malaria episode was defined as an axillary temperature of ≥37.5°C or history of fever in the past 24 hours and *P. falciparum* trophozoite count >2,500 parasites/μl. Splenomegaly of any size was identified by Hackett’s classification [26]. Haemoglobin was measured using a HemoCue machine and reported as g/dl. Child age was defined as age (years) at enrolment.

### Statistical analysis

Demographic characteristics (age, sex and health district) and season-specific malariometric indices were compared among normal (AA) and abnormal Hb genotypes using Kruskal-Wallis tests for continuous and Chi-square or Fischer’s exact tests for categorical measures.

Clinical malaria incidence was calculated within strata defined by commonly occurring (AA, AC, and AS) genotype and season of follow-up as the total number of clinical malaria episodes observed by active and passive surveillance divided by the total number of person-years at risk. Children were excluded from incidence analysis for 28 days after recording an episode, to ensure that the infection causing the episode was only recorded once. The study period ended 365 days after the first visit or on the date of death/emigration from the study locale (N = 2).

Age-specific risks associated with Hb genotype were evaluated using a Poisson regression model of haemoglobin genotype as a predictor of rate of malaria episodes observed over one year follow-up. The model was used to estimate rate ratios associated with AC, AS or either genotype relative to AA within age strata after adjustment for health district. Analyses were repeated among the full cohort to formally test for interactions of AC and AS genotype with age group. Cumulative incidence of clinical malaria within genotype strata was estimated using the Kaplan-Meier method. Malaria-free survival curves were compared among genotypes using the log-rank test.

All data were double entered in Epi Info Version 6.04 (CDC, Atlanta, USA) and statistical analyses were performed in SAS Version 9.2.

### Ethical processes

The study protocol was explained to the local population prior to study initiation. Written informed consent was obtained from the parents or guardians of all participating children before enrolment. The protocol of this study was approved by the Burkina Faso Health Research Ethics Review Committee.

## Results

### Baseline characteristics of the study cohort

The study cohort was divided into five predefined age groups: nought to one year (*n* = 75), one to two years (*n = 93*), two to three years (*n = 97*), three to four years (*n = 102*), and four to five years (*n = 85)*. Mean age of the cohort was 32 months; 47.8% were female (Table 1). Frequencies of each haemoglobin genotype were 73.2% AA; 15.0% AC; 8.2% AS; 2.2% CC; 1.1% CS and 0.2% SS. Age and sex distributions were similar among genotype groups.

**Table 1:** Demographic characteristics by Age, sex and haemoglobin genotype

**Haemoglobin type**		**Gender**		**All**
		**Male**	**Female**	
AA	N	175	156	331
	Age (months), mean	33.2	32.0	32.6
	Age (months), SD	17.1	15.9	16.5
AC	N	33	35	68
	Age (months), mean	29.0	29.5	29.3
	Age (months), SD	18.0	17.2	17.5
AS	N	18	19	37
	Age (months), mean	31.3	32.7	32.0
	Age (months), SD	11.7	16.5	14.2
CC	N	6	4	10
	Age (months), mean	19.1	17.1	18.3
	Age (months), SD	17.9	17.2	16.6
SC	N	4	1	5
	Age (months), mean	41.7	1.7	33.7
	Age (months), SD	15.4	0	22.3
SS	N	1	0	1
	Age (months), mean	43.6	0	43.6
	Age (months), SD	0	0	0
Non-AA	N	62	59	121
	Age (months), mean	29.8	29.2	29.5
	Age (months), SD	16.4	17.3	16.8

Note: Age and sex distribution were similar among genotype.

### Malariometric indices

Prevalence of *P. falciparum* infection decreased from 59.7% to 50.4% from the low to high season; geometric mean parasite density increased from 1,579 (1,315–1,896) to 2,748 (2,104–3,589) trophozoites/μl. Prevalence of clinical malaria varied minimally (11.3% to 12.9%) (Tables 2 and 3). Clinical malaria prevalence and mean haemoglobin did not vary by Hb genotype within either season; however *P. falciparum* infection was less prevalent among children with CC relative to AA genotype at each survey (p < 0.05 for each).

**Table 2:** Malariometric indices by haemoglobin genotype

**Infection characteristic**	**AA (N = 331)**	**AC (N = 68)**	**AS (N = 37)**	**CC (N = 10)**	**SC (N = 5)**	**SS (N = 1)**	**All (N = 452)**
Symptomatic malaria (fever and >0 parasites/μl), N (%)	39 (11.8%)	7 (10.3%)	4 (10.8%)	1 (10.0%)	0 (0.0%)	0 (0.0%)	51 (11.3%)
*P. falciparum* infection (>0 parasites/μl), N (%)	205 (61.9%)	39 (57.4%)	20 (54.1%)	2 (20.0%)*	4 (80.0%)	0 (0.0%)	270 (59.7%)
Symptomatic malaria (fever and >2500 parasites/μl)	22 (6.6%)	3 (4.4%)	0 (0.0%)	0 (0.0%)	0 (0.0%)	0 (0.0%)	25 (5.5%)
*P. falciparum* infection (>2500 parasites/μl), N (%)	82 (24.8%)	15 (22.1%)	6 (16.2%)	1 (10.0%)	1 (20.0%)	0 (0.0%)	105 (23.2%)
Gametocyte carriage, N (%)	101 (30.5%)	21 (30.9%)	10 (27.0%)	3 (30.0%)	3 (60.0%)	0 (0.0%)	138 (30.5%)
Splenomegaly, N (%)	67 (20.2%)	9 (13.2%)	4 (11.1%)	1 (10.0%)	0 (0.0%)	0 (0.0%)	81 (18.0%)
Haemoglobin (g/dl), mean (SD)	9.7 (1.5)	9.5 (1.6)	9.5 (1.5)	9.4 (1.5)	11.1 (0.8)	10.1	9.6 (1.5)
*P. falciparum* density (per/μl), GM (95% CI)	1546 (1259,1898)	1904 (1159, 3128)	1305 (527, 3231)	3088 (51, 185428)	1418 (104,19382)	---	1579 (1315, 1896)
Gametocyte density (per/μl), GM (95% CI)	51 (41, 64)	48 (30, 75)	95 (54, 166)	81 (2, 3110)	22 (0, 62183)	---	52 (43, 64)

Low transmission season.

*P for difference vs AA < 0.05.

**P for difference vs AA < 0.01.

**Table 3:** Malariometric indices by haemoglobin genotype

**Infection characteristic**	**AA (N = 305)**	**AC (N = 64)**	**AS (N = 35)**	**CC (N = 9)**	**SC (N = 5)**	**SS (N = 1)**	**All (N = 419)**
Symptomatic malaria (fever and > 0 parasites/μl), N (%)	37 (12.1%)	13 (20.3%)	2 (5.7%)	0 (0.0%)	2 (40.0%)	0 (0.0%)	54 (12.9%)
*P. falciparum* infection (> 0 parasites/μl), N (%)	152 (49.8%)	34 (53.1%)	19 (54.3%)	1 (11.1%)*	4 (80.0%)	1 (100.0%)	211 (50.4%)
Symptomatic malaria (fever and >2500 parasites/μl)	27 (8.9%)	9 (14.1%)	1 (20.0%)	0 (0.0%)	0 (0.0%)	0 (0.0%)	37 (8.8%)
*P. falciparum* infection (> 2500 parasites/μl), N (%)	84 (27.5%)	22 (34.4%)	4 (11.4%)*	0 (0.0%)	2 (40.0%)	1 (100.0%)	113 (27.0%)
Gametocyte carriage, N (%)	60 (19.7%)	11 (17.2%)	3 (8.6%)	2 (22.2%)	0 (0.0%)	0 (0.0%)	76 (18.1%)
Splenomegaly, N (%)	26 (8.5%)	7 (10.9%)	2 (5.7%)	0 (0.0%)	0 (0.0%)	0 (0.0%)	35 (8.4%)
Haemoglobin (g/dl), mean (SD)	10.2 (1.5)	9.9 (1.4)	9.9 (1.5)	10.0 (1.5)	11.1 (1.1)	10.5	10.1 (1.5)
*P. falciparum* density (per/μl), GM (95% CI)	2690 (1978, 3659)	5823 (3117, 10878)*	986 (383, 2541)*	364	1632 (7, 375567)	9740	2748 (2104, 3589)
Gametocyte density (per/μl), GM (95% CI)	41 (31, 53)	20 (11, 37)*	23 (3, 173)	34 (4, 263)	(NA)	(NA)	36 (28, 45)

High transmission season.

*P for difference vs AA < 0.05.

**P for difference vs AA < 0.01.

### Incidence of clinical malaria

Incidence of clinical malaria (95% CI) among the full cohort was 1.8 (1.7-1.9) episodes per person-year after one year follow-up. The season-specific incidence rate was 3.1 times higher in the high relative to the low transmission season (Table 4). Within each season incidence rates were higher among children with AA genotype relative to abnormal genotypes, although differences were not statistically significant.

**Table 4:** Incidence of malaria episodes (fever and parasitemia >2500/μl) by haemoglobin genotype and season

**Season**	**Haemoglobin genotype**	**Number of children**	**Number of episodes**	**Mean number of episodes per child**	**Total person- years**	**Incidence rate (95% CI) (per person-year)**
Low	AA	331	184	0.6	177.0	1.0 (0.9, 1.2)
	AC	68	31	0.5	37.1	0.8 (0.6, 1.2)
	AS	37	15	0.4	20.4	0.7 (0.4, 1.2)
	CC	10	1	0.1	5.8	0.2 (0.0, 1.0)
	SC	5	1	0.2	2.8	0.4 (0.0, 2.0)
	SS	1	0	0.0	0.6	0.0 (0.0, 6.4)
	Total	452	232	0.5	243.6	1.0 (0.8, 1.1)
High	AA	305	363	1.1	112.4	3.2 (2.9, 3.6)
	AC	64	74	1.1	23.0	3.2 (2.5, 4.0)
	AS	35	35	0.9	13.1	2.7 (1.9, 3.7)
	CC	9	8	0.8	3.7	2.2 (0.9, 4.3)
	SC	5	5	1.0	1.8	2.9 (0.9, 6.7)
	SS	1	0	0.0	0.4	0.0 (0.0, 8.8)
	Total	419	485	1.1	154.4	3.1 (2.9, 3.4)
Low and High	AA	331	542	1.6	288.0	1.9 (1.7, 2.0)
	AC	68	104	1.5	59.8	1.7 (1.4, 2.1)
	AS	37	50	1.4	33.1	1.5 (1.1, 2.0)
	CC	10	9	0.9	9.3	1.0 (0.4, 1.8)
	SC	5	6	1.2	4.5	1.3 (0.5, 2.9)
	SS	1	0	0.0	1.0	0.0 (0.0, 3.7)
	Total	452	711	1.6	395.7	1.8 (1.7, 1.9)

Note: Incidence of malaria episodes was calculated as the number of malaria cases.

divided by total person-years (p-yrs) at risk).

In age-stratified analyses adjusted for health district, AC genotype was associated with lower incidence of clinical malaria relative to AA among children aged 1–2 years [rate ratio (95% CI) = 0.66 (0.42, 1.04); p = 0.07] and 2–3 years [rate ratio (95% CI) = 0.37 (0.18, 0.75); p = 0.01] (Table 5). An association of opposite direction was however apparent among children aged 3–4 years: rate ratio (95% CI) = 1.61 (1.08, 2.41); p = 0.02. An association of AS genotype with lower incidence of clinical malaria relative to AA approached significance among children aged 2–3 years: rate ratio (95% CI) = 0.63 (0.40, 1.101); p = 0.06.

**Table 5:** Incidence of malaria episodes (fever and parasitemia >2500 parasites/μl) by haemoglobin genotype and age at enrollment

**Age**	**Haemoglobin genotype**	**Number of children**	**Number of episodes**	**Incidence rate (95% CI) (per person-year)**	**District-adjusted rate ratio (95% CI)**	**P value**
0-1 yrs	AA	52	115	2.7 (2.2, 3.2)	---	---
	AC	15	31	2.5 (1.7, 3.5)	0.93 (0.62, 1.38)	0.72
	AS	2	2	1.1 (0.1, 3.9)	0.41 (0.10, 1.65)	0.21
1-2 yrs	AA	66	131	2.3 (2.0, 2.8)	---	---
	AC	16	22	1.5 (1.0, 2.3)	0.66 (0.42, 1.04)	0.07
	AS	9	19	2.5 (1.5, 3.9)	0.93 (0.56, 1.54)	0.77
2-3 yrs	AA	70	145	2.5 (2.1, 2.9)	---	---
	AC	10	8	0.9 (0.4, 1.7)	0.37 (0.18, 0.75)	**0.01**
	AS	14	20	1.6 (1.0, 2.5)	0.63 (0.40, 1.01)	0.06
3-4 yrs	AA	77	95	1.4 (1.1, 1.7)	---	---
	AC	17	32	2.2 (1.5, 3.1)	1.61 (1.08, 2.41)	**0.02**
	AS	6	9	1.7 (0.8, 3.2)	1.05 (0.53, 2.09)	0.89
4-5 yrs	AA	66	56	0.9 (0.7, 1.2)	---	---
	AC	10	11	1.2 (0.6, 2.2)	1.27 (0.66, 2.44)	0.48
	AS	6	0	0.0 (0.0, 0.6)	0.00	1.00

In analyses inclusive of all children aged 1–3 years and adjusted for health district, presence of either AS or AC genotype was associated with reduced risk of similar magnitude: rate ratio (95% CI) = 0.63 (0.49, 0.83); p = <0.001. Among the full cohort, risk associated with AC genotype varied significantly among children younger versus older than three years after adjustment for health district (p for interaction < 0.001).

**Figure 2:**
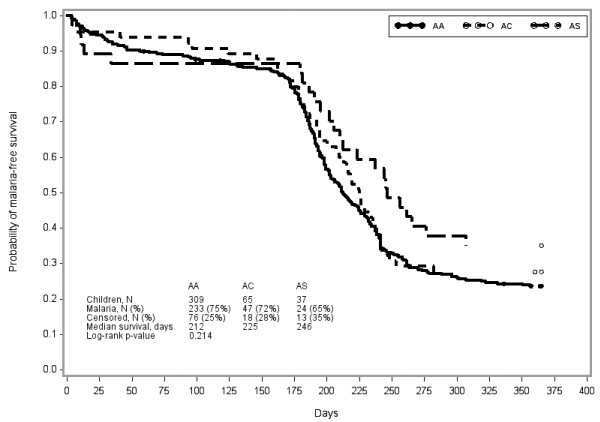
Time to first malaria episode by haemoglobin abnormality among children aged 0–5 years, over one year follow-up (low transmission season and high transmission season).

**Figure 3:**
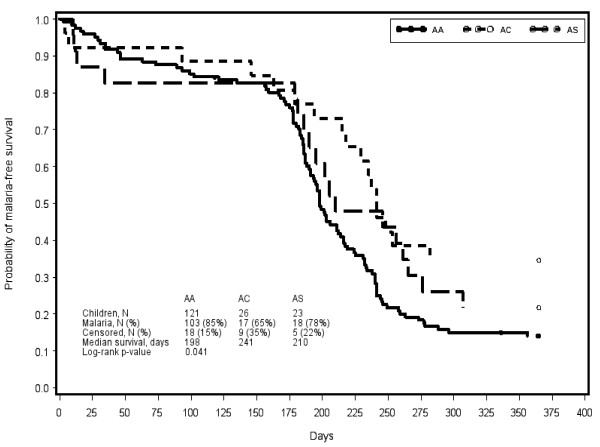
Time to first malaria episode by haemoglobin abnormality among children aged 1–3 years, over one year follow-up (low transmission season and high transmission season).

Time to first malaria episode did not significantly differ by AA, AC or AS genotype among the full cohort (p = 0.21) (Figure 2). Among children aged 1–3 years, however, delayed first malaria was apparent among children with AC (241 days) or AS (201 days) genotype relative to AA (198 days) (p = 0.04) (Figure 3). Associations of genotype with time to first malaria were not apparent among other age groups.

## Discussion

In this cohort of children under five years of age living in a high malaria-transmission region of Burkina Faso, AC or AS Hb genotype was associated with lower risk of clinical malaria relative to the AA genotype among children aged one to three years. This association was attenuated though approached significance among the full cohort. These data suggest that Hb genotype should be considered a potentially important confounder, particularly among younger children, in evaluations of clinical malaria risk in endemic regions.

The haemoglobin S and C genes occurred in the cohort with similar frequencies to those previously published [17,27,28]; 26% were carriers of either HbS or HbC. The mechanism by which HbS protects against malaria has been the subject of speculation for more than 50 years. While protection may largely be conferred by the physical characteristics of HbS erythrocytes, a number of studies suggest that HbAS may also enhance the acquisition of naturally acquired immunity [19,29]. Associations between the HbC trait and malaria risk, however, have not been uniformly established [30].

The finding that parasitaemia prevalence varied only minimally among AA, AC and AS genotypes in the full cohort is consistent with previous studies conducted in various parts of Africa [9,31-33]. Malaria premunition has also been found comparable among haemoglobin groups among patients greater than six years of age [30]. Age-specific associations of malariometric indices with Hb gentoypes may be hypothesized to reflect the development of anti-malarial immunity [30] as the protective action of haemoglobin may be masked by maternally transmitted antibodies among children less than six months of age [34]. While age-specific patterns of association were not apparent in the current cross-sectional measures, further inferences are limited due to the small number of children within age and genotype strata.

Mean parasite density was markedly lower in children AS relative to AA genotype normal haemoglobin, consistent with previous reports [17,27]. Indeed, abnormal haemoglobin may not allow for optimal development of *Plasmodium* in deep organs where oxygen pressure is reduced. Parasite density was however higher among AC relative to AA genotype, suggesting potential mechanistic variation among protection afforded by abnormal genotypes in early childhood. Malaria-risk reduction associated with HbAS genotype has been reported in Mali[27,35] Burkina [17], Ghana [36] and Kenya[11]; a similar protective advantage of HbAC has been less consistently supported.

Abnormal Hb genotype in this cohort was further associated with lower incidence of clinical malaria episodes and delayed first episode among children aged one to three years. These effects were more subtle in infants and older children. Several studies have identified a protective effect of abnormal Hb against clinical malaria [17,35]. Although HbAS was found unassociated with time to first malaria episode in Gabon [37], HbAS was associated with reduced time to all-cause mortality in Kenya, an effect attributed to reduction in malaria-specific outcomes including severe malarial anaemia and high-density parasitaemia[38]. Proposed mechanisms of protection [39] include decreased red blood cell invasion or poor growth under low-oxygen tension[40]; and accelerated acquisition of antibodies specific for *P. falciparum* erythrocyte membrane protein-1 (PfEMP-1) and other variant surface antigens[41].

Alternative findings among cohorts may in part reflect variation in age distribution and associated development of acquired immunity, malaria endemicity, and administration of anti-malarial drugs. Estimates of malaria burden in the current study are likely underestimates, due to prompt treatment of malaria episodes identified by active surveillance. This study is further limited by the small number of children with abnormal genotypes and restriction of follow-up to one year, preventing stronger inferences related to changing risk associated with alternative genotypes throughout early childhood.

## Conclusions

In this cohort of children under five years of age, AC or AS Hb genotypes was associated with lower risk of clinical malaria relative to normal genotype among children aged one to three years. This age-specific association may suggest influences of HbC and HbS genotypes in the development of naturally acquired immunity in early childhood. Evaluations of anti-malarial interventions in endemic regions should consider Hb genotype as a potentially important confounder, particularly among young children.

## Abbreviations

AA: Wild type; SS: Sickle cell; AS: Sickle trait; AC: Heterozygous for haemoglobin C; CC: Homozygote for haemoglobin C; SC: Haemoglobins S and C; Non-AA: Abnormal haemoglobin (AS, AS, SC, SS and CC); PCR: Polymerase chain reaction.

## Competing interest

The authors declare that they have no competing interests.

## Authors’ contributions

ECB designed the study, collected data and coordinated the study, performed statistical analysis and wrote the first draft of the manuscript. SBS, AT, IN, AO and ATK coordinated and participated in the design of the study, participated in the statistical analysis and procedures and the drafting of the manuscript. EBC, IS, SS and AD participated in the laboratory work and data interpretation. JBY, OE and ECB carried out the study. NW, MS and TJTD contributed to analysis and interpretation and to contribute to writing the paper. All the authors read and approved the final version.
